# *Blesser* relationships among orphaned adolescent girls in contexts of poverty and gender inequality in South African townships

**DOI:** 10.1371/journal.pone.0299190

**Published:** 2024-10-17

**Authors:** Ndumiso Daluxolo Ngidi, Xolani Ntinga, Ayanda Tshazi, Relebohile Moletsane

**Affiliations:** 1 School of Education, University of KwaZulu-Natal, Durban, South Africa; 2 Centre for Community-Based Research, Human Sciences Research Council, Pietermaritzburg, South Africa; University of KwaZulu-Natal College of Health Sciences, SOUTH AFRICA

## Abstract

The term *blesser* has become part of South Africa’s contemporary lexicon, replacing the older terminology of ‘sugar daddy.’ While much recent literature has focused on the *blesser* phenomenon, the voices of orphaned adolescent girls on their entanglement in *blesser* relationships have had insufficient attention. Using the theory of gender and power as an analytical lens, this qualitative study analyses the visual and textual data generated by orphaned adolescent girls on their relationships with *blessers*. To generate data, the participants used photovoice to represent their relationships with older male sexual partners in their resource-poor South African township neighbourhoods. Our analysis reveals a set of factors that render orphaned adolescent girls vulnerable to age-disparate relationships, such as the structural dimensions of their lives, including their status as orphaned girls, heteropatriarchy, age-based hierarchies, and poverty in their households and communities. On the other hand, our analysis explores the less understood area of the relative agency, intentionality, and proactive approach that orphaned girls take to initiating and negotiating *blesser* relationships. The findings have implications for further research that will expand our understanding of girls’ agency—and the structural limits to that agency—in adverse socio-cultural circumstances. Such research holds potential for interventions that might enable orphaned girls to better advocate for themselves in the context of unequal power relations.

## Introduction

*Well with me, let me just say my situation is very bad (referring to poverty at home). I ended up dating a man that is older than me. It ended up like that because it happens that he buys me nice things, and gifts and also gives me money and I end up sleeping with him even though I don’t want to*

(16-year-old Lwandle)

Lwandle (not her real name) is a 16-year-old orphaned girl who lives with her extended family in a poorly-resourced township near Durban in South Africa. Explaining her intimate sexual encounters, she points to a context characterized by poverty in her household and her reliance on an older male sexual partner for financial and material support. She acknowledges the precarity of her relationship in that she cannot refuse sex with her partner lest she loses the security it provides. This is not surprising as Lwandle lives in a township where poverty is entrenched, gendered, and rooted in a rigid system of heteropatriarchy. Her situation is worsened by the fact that she lost her biological parents and, as a double orphan, depends on an equally impoverished extended family that struggles to provide her with necessities. These conditions render girls like her, who are adolescent orphans, susceptible to sexual and economic exploitation [1].

Lwandle’s narrative highlights the perilous state of orphaned girls’ lives and their entanglement with older male sexual partners in contexts of gender inequality and poverty in South African townships. While public health research has tended to construct girls’ sexual involvement with older men from the context of HIV, gender-based violence, and other poor health outcomes [2, 3], we approach our analysis of these age-disparate sexual relationships from the perspectives of the orphaned adolescent girls themselves.

African scholars have highlighted the plight of orphaned girls in high-risk and impoverished neighbourhoods and households with limited resource access [4]. One of the reasons is that in such contexts, extended families, and sometimes neighbours, struggle to provide for these children due to limited financial resources and essentials [5–7]. Moreover, the loss of a parent is a risk factor for orphaned girls’ adverse outcomes, including neglect, abuse, and exploitation [1, 8]. Salifu’s research also suggests that orphaned girls often grow up in unsafe and exploitative environments that include sexual coercion and violence [9] and have few opportunities to develop and little protection from abuse and maltreatment [1]. As Save the Children 2019 reports [10], as a result, orphaned girls are more likely to struggle to access education, care, and support than non-orphaned girls. These children often grow up without access to a significant adult role model and a solid tie to an extended family that might provide care, support, and sanctuary [11]. Research continues to highlight adolescent girls’ sexual relations with older men, mainly for survival and material benefits [2]. Missing from this literature are studies that focus on these relationships from the perspectives of orphaned adolescent girls and on their agency, however, limited, in such encounters. This article explores this knowledge gap.

### Age-disparate relationships in contexts of poverty and gender inequality

Studies on adolescent sexualities in South Africa burgeoned within the context of the post-apartheid government’s priority to curb the spread and impact of HIV & AIDS, other sexually transmitted infections (STIs), gender violence (including rape and femicide), and unplanned teenage pregnancies [12–16]. These multiple epidemics have been exacerbated by the country’s struggle with socioeconomic inequality and poverty which tends to be negatively skewed against the black majority. Due to unequal gender norms in communities, such poverty and inequalities are even more pronounced among black African girls and women who live mainly in rural and township settings. These communities are characterized by poverty, food insecurity, low-quality health and education institutions, high unemployment rates, crime, and violence [17]. For example, in Inanda, Ntuzuma, and Kwamashu (INK), the neighbourhoods where this study was located, poverty, food insecurity, and unemployment are rife, with around 40% of the population being unemployed, and 75% of all households earning less than R10 000 (US$560) per annum [18]. These townships are in KwaZulu-Natal (KZN), a province with over 53% of its population living below the poverty threshold. Close to half of the households in the province are categorized as low-income households, with an annual income below R55 000 (US$ 3 781). KZN is also the country’s HIV epicentre, and adolescent girls and young women (AGYW) are most vulnerable to infections [19, 20]. South Africa offers social relief programs through a social grant system to curb poverty shocks and food insecurity. However, these grants are hardly enough to pull households out of poverty.

Our research examines adolescent orphaned girls’ narratives about engaging in age-disparate transactional sexual relationships in this resource-poor context. Such age-disparate relationships (where the male partner is up to 10 years older or more) [21] are a global practice in which AGYW access resources from older men in exchange for sex [22, 23]. These relationships are the focus of various studies, with some researchers describing them as sexual relationships between older men and AGYW [24] in exchange for money or gifts [25].

In South Africa, the concept of *blesser* relationships emerged as a popular colloquial term for such age-disparate sexual relationships [26]. The term emerged around 2016 after a series of viral social media posts of young women purporting to have anonymous sponsors who ‘blessed’ them with expensive, and often luxurious, gifts and shopping trips around the globe [24]. Within this context, a *blesse*r is an older man who provides gifts, material, or financial support to an AGYW in exchange for sex. *Blesser* has become part of South Africa’s contemporary lexicon that is repackaged to replace the older terminology of ‘sugar daddy.’ The ‘beneficiaries’ of a *blesser’s* gifts are called the ‘*blessee*’ or the ‘blessed.’

While these relationships receive social condemnation and *blesser* relationships remain taboo in several communities [27], they continue persistently across Africa [2, 19–22]. For example, one study [28] on the sexual practices of AGYW found that 41% of young women aged 15–24 years were involved in sexual relationships with older men. In KwaZulu-Natal, researchers [29] have found that 38% of AGYW from rural settings reported having a sexual partner five or more years older than themselves. Furthermore, other researchers [25] report that around 35% of the sampled AGYW were in relationships with men who were six years or older. Similar trends have been reported in other African contexts. For example, in Zimbabwe’s Manicaland Province, 65% of AGYW reported partner age differences of at least five years [30], while in Uganda 30% of AGYW in the Rakai Community Cohort Study reported having a male partner of at least 5 years older [31].

As one of the well-known drivers of new HIV infections among AGYW aged 15–24 years in Sub-Saharan Africa, *blesser* relationships have received increasing attention in public health discourses in African global south contexts in the last 20 years [3, 32, 33]. As studies suggest, the incidence and prevalence of HIV among adolescent girls in South Africa have reached crisis proportions [2]. These relationships are characterized by an imbalance of power occasioned by age and gender inequalities, economic disparities, and violence or threats [3]. AGYW are often not able to negotiate safer sex practices with their older male partners and are vulnerable to multiple forms of sexual exploitation and violence [33]. In response, several initiatives targeting adolescents’ sexual and reproductive health and rights (SRHR) have focused on the vulnerability of AGYW to sexual relationships with older men. While such initiatives exist (see [34], little has been achieved to curb *blesser* sexual partnerships [35]. Indeed, research evidence points to adverse health and educational outcomes associated with transactional sex [19, 36]. Yet, studies on how and why orphaned AGYW engage in *blesser* relationships remain non-existent.

### Analytical framework

We locate our analysis of these complex entanglements within Connell’s [37] theory of gender and power. The theory argues that three social structures characterise relationships between men and women: 1) the sexual division of labour, 2) the sexual division of power, and 3) social norms and affective attachments around femininity and masculinity. According to Connell, these structures are present and interact at the household, societal, and institutional levels, creating, as they do, gender inequities across all domains of women’s lives. Thus, the presence and interaction of these structures produce and reproduce gender inequalities that operate to subordinate women and fuel their vulnerability in various spheres of life. The sexual division of labour often limits AGYW to domestic roles that limit their economic prospects, thus creating financial dependencies on men, and consequently gendered poverty. In many poor communities, this often drives AGYW to seek some form of economic security in age-disparate sexual relationships [38]. These relationships further entrench and reinforce the sexual division of power through hegemonic social mechanisms that include male sexual conquest, coercion, control, and authority within heterosexual relationships. Because gender differences in the division of power “are an important determinant of the outcome of sexual negotiations” [39], in the context of *blesser* relationships, AGYW are less likely to have the negotiating upper hand [40]. In addition, social norms and expectations enforce strict gender roles on women and men and dictate how they express their sexuality.

In the context of this study, older men engage in transactional sexual relationships with AGYW due to hegemonic norms of masculinity and their need to assert dominance and status through the provision of resources [41]. AGYW in turn tend to embrace and submit to a norm of femininity that places them at risk for sexual exploitation and abuse. The theory of gender and power provides a framework to explain how AGYW negotiate dating relationships. In this study, as Connell’s theory maintains, we understand the context of *blesser* relationships as involving gender and power inequalities, which subordinate orphaned girls and force them to rely on older men for material and economic sustenance while at the same time acknowledging them as agentic and active participants in the relationship. We use the theory here to analyse the complex factors that intersect to influence the orphaned adolescent girls’ experiences and agency (or lack thereof) in these age-disparate sexual relationships with older men.

## Methodology

### Approach

Ethical approval to conduct this research was granted by the University of KwaZulu-Natal College of Humanities and Social Sciences Research Ethics Committee (HSS/1764/016D) on 06 March 2017. The KwaZulu-Natal Department of Education provided permission to approach and recruit orphans from a school in the province (REF: 2/4/8/1149) on 1 February 2017. Data collection took place between March to October 2017. To access the participants, we negotiated and received written informed consent from the participants’ primary caregivers and the school’s management. The research team detailed the research to the participants, then after the participants provided written assent for their participation. The participants also provided written consent for the use of their visual data, such as photographs for publication and other research purposes. Since this study worked with children deemed vulnerable, and because of the sensitive nature of the topic of CSA, we negotiated and received support from two social workers who provided psychosocial services to the participants if and when they needed them. Further, since the South African Children’s Act 38/2005 stipulates that cases of child abuse be reported to social authorities, the emerging narratives of violence were discussed with the social workers. The participants did consult (voluntarily) with the social workers and we trust that as trained professionals in the service of child welfare, the social workers were capable of advising the participants or acting accordingly.

### Study context

The study was conducted in three neighbouring township communities in the greater eThekwini Municipality, whose central business district is the port city of Durban. eThekwini Municipality has sub-Saharan Africa’s largest and busiest seaport [42]. The townships where our study was based share similar socioeconomic characteristics including, among others, being among the country’s most densely populated, having high youth unemployment rates, and high levels of poverty. Poverty has been described as a significant determinant for girls dating older men [38]. For example, around 54% of South African adolescents live below the poverty line of R671 (US$46) per month [43]. Available research [44] suggests that socioeconomic challenges that are associated with poverty (i.e., low-cost or informal housing, low-quality healthcare and education provision, resource-poor neighbourhoods, and dysfunctional interpersonal and family dynamics) are some of the factors that contribute to adolescent girls’ increased risk of violence within sexual relationships. For adolescent girls, growing up in chronically poor contexts is complicated because their bodies are sexualized as they begin to mature physically. Moreover, poverty in the country is gendered and has the greatest impact on girls and women who live in rural and township settings [45]. It is within this broader context that the orphaned girls who participated in this study lived, and within which our analysis focuses.

### Participants and data generation

This paper is premised on our understanding that if interventions are to respond effectively to age-disparate sexual relationships and their impact, research must prioritise the voices of the AGYW on these relationships and the socio-economic factors that drive them. Yet, despite the view that girls assume a powerless position when they engage in *blesser* relationships [3], literature is relatively silent on the perspectives that orphaned adolescent girls have on their experiences of *blesser* relationships, and the power dynamics in these. Furthermore, the transition to adulthood gives adolescents many opportunities to experiment and exercise autonomy over their sexual lives. This, in turn, opens avenues for girls to choose (or even be coerced into) sexual partnerships. However, orphaned girls are insufficiently studied in these relationships. The data for this paper is drawn from a larger participatory inquiry with 14 adolescent girls identified as double orphans (aged 13–17 years) who were purposively selected from a secondary school in the INK precinct. The larger study examined these girls’ understandings of, as well as their vulnerability and experiences with, sexual violence in and around their schools. Participants were selected because they had lost both of their biological parents. Due to the sensitive nature of the study, we took care to ensure that data generation was done responsibly and that the participants were empowered rather than victimized. Two social workers were enlisted in the study to offer support to participants who might experience distress.

The study used participatory visual methods to engage these orphaned adolescents in reflection on their understandings and experiences of sexual violence. This article reports data generated from a day-long photovoice workshop, which was augmented by a focus group discussion. Photovoice is a research method in which participants are given cameras to document their everyday realities of the phenomenon under study [46–49]. This technique has since been hailed for its resourcefulness in engaging children and young people in sensitive topics in a relaxed way [50]. Using photovoice and focus group discussion in the study enabled participants to feel safe and to amplify their voices. Secondly, it enabled participants to relate their social issues to their specific context. Thirdly, it gave participants the freedom to decide on the aspects of the research topic that mattered to them. Finally, in using photovoice, the participants decided what they felt was important to discuss with the research assistant.

Participants were asked to work in pairs using this prompt: *take photos showing what vulnerability to sexual violence looks like in and around your school environment*. However, as the research progressed, while the larger group of girls were discussing their visual depictions of vulnerability to sexual violence, two pairs of girls presented two pictures that illustrated the context, dynamics, and orphaned girls’ engagement in *blesser* relationships, which triggered the need to examine these experiences and perspectives in more depth. Thus, we invited all the girls in the group to engage in a focus group discussion to explore their perspectives on *blesser* relationships; a step to which they verbally consented given that they had already provided written consent. For this paper, we analyse the two photos and the transcripts of the focus group discussion that ensued. An experienced black African woman researcher in her late twenties was hired to facilitate the focus group discussion and to create a safe environment for the participants to discuss their perspectives.

All the participants were present during the group discussion, which was conducted in isiZulu, the local language of the participants. The discussion ran for approximately two hours, was audio-taped, and later transcribed verbatim. The transcript was later translated into English for analysis. To ensure accurate reporting of participants’ meaning-making and views during translation, we read and re-read the transcripts, since three of the authors are isiZulu first-language speakers. Further, an independent language practice graduate student was hired to read and confirm the translations. We used thematic analysis to analyse the transcript from the focus group discussion. Data were first coded into broad themes, and a thematic approach helped to organize the data into themes that helped to organize the findings.

## Findings

### Being an orphaned adolescent girl in a *blesser* relationship

As stated above, our findings are drawn from a larger study that examined orphaned adolescent girls’ vulnerability to sexual violence. This specific study explores the context and dynamics of orphaned adolescent girls in their relationships with *blessers*. The findings yielded six themes that are discussed below.

#### Male economic capital and orphaned girls’ vulnerability

**Fig 1:**
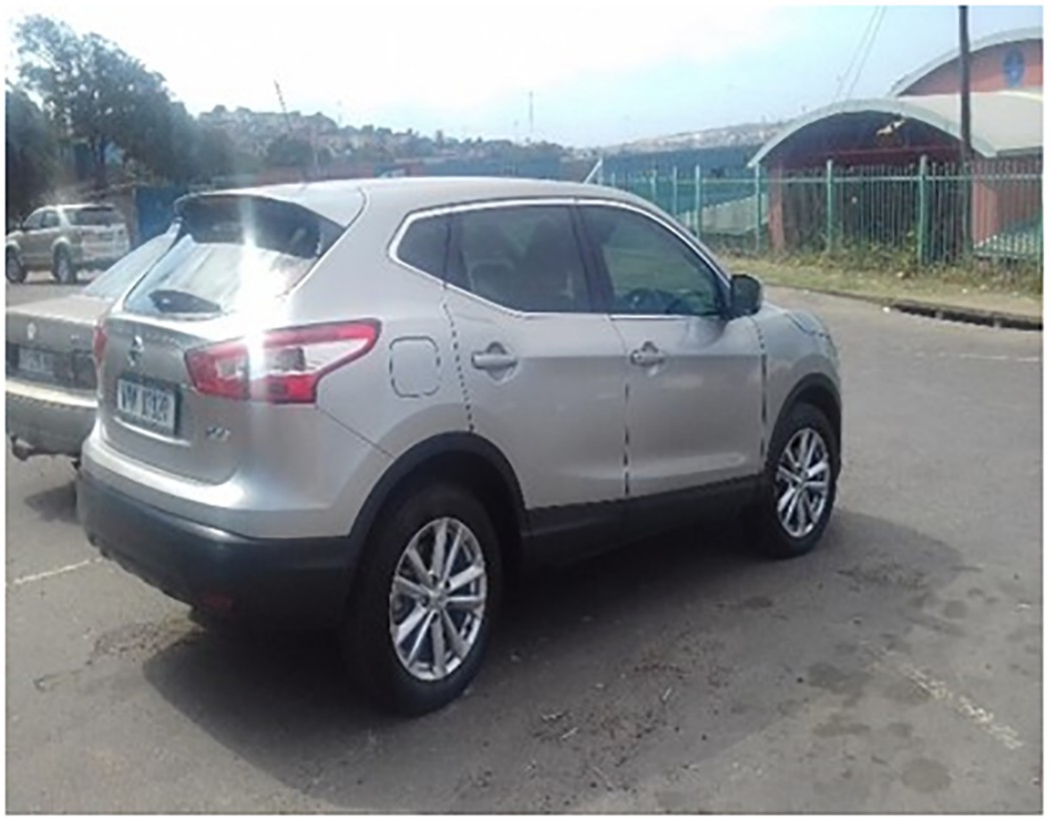
A *blesser’s* car.

The data analysed in this article emerged when two participants fortuitously presented photovoice images they had produced during a focus group discussion. The first image (Fig 1) shows an expensive car that the respondents suggested belonged to a *blesser*.

In the accompanying description of their image, the participants wrote that “*blessers are older men who have money*, *love younger girls*, *and even sexually use us (girls)*. *They (blessers) live in our communities*.” Considering the description of *blessers* as “*older men who have money*”, three things are notable here. First, this finding suggests that girls use age and expensive possessions to determine a man’s capacity to ‘bless’ them; that is, to provide for the needs that their families cannot meet. While the girls acknowledge *blessers* as older men who have money and who “*love to have sex with younger girls*,” they had some criteria for identifying a man who is eligible to be a *blesser*. To be eligible, such men had to have economic capital, revealed through ownership of an expensive car symbolising the economic status that *blessers* use to bargain entry into relationships with girls. In resource-constrained contexts where families lack economic resources, an expensive vehicle represents economic capital that *blessers* leverage for negotiating heterosexual relationships with girls.

In other words, *blessers* leverage girls’ desire for financial security and material needs and use this information to enter sexual relationships with orphaned girls. In turn, girls here are attracted to transactional relationships with older men, not for luxury or glamour, as previous research reports (see, for example, [51]), but to meet their everyday material needs. The following excerpt from the FGD illustrates:

*You are forced to date a blesser because some of our relatives do not care about our needs like buying us sanitary pads, deodorant, all the important stuff, and face wash. All the things that are important for girls. You see, for some of us at home, they do not care about that*

*(15-year-old Tee)*.

Tee confirms that a family’s inability to provide a girl’s basic hygiene (and other) needs influences the decision to have a *blesser*. Therefore, contrary to dominant narratives in the literature [51], for her, it is not a desire for luxury but the need for material provision that informs *blesser* relationships among orphaned girls like her in this study.

Third, the participants’ assertion that *blessers* “*even sexually use us*” suggests a context of sexual exploitation. This indicates an awareness of the transactional nature of girls’ relationships with older men and the unequal power relations that characterise them. The girls make judgments based on criteria (age and possessions) that the men will be able to meet their material needs. Participants displayed an understanding that older men who sleep with young girls use their financial power to access their bodies sexually in return for material provisions that their families do not meet.

**Fig 2:**
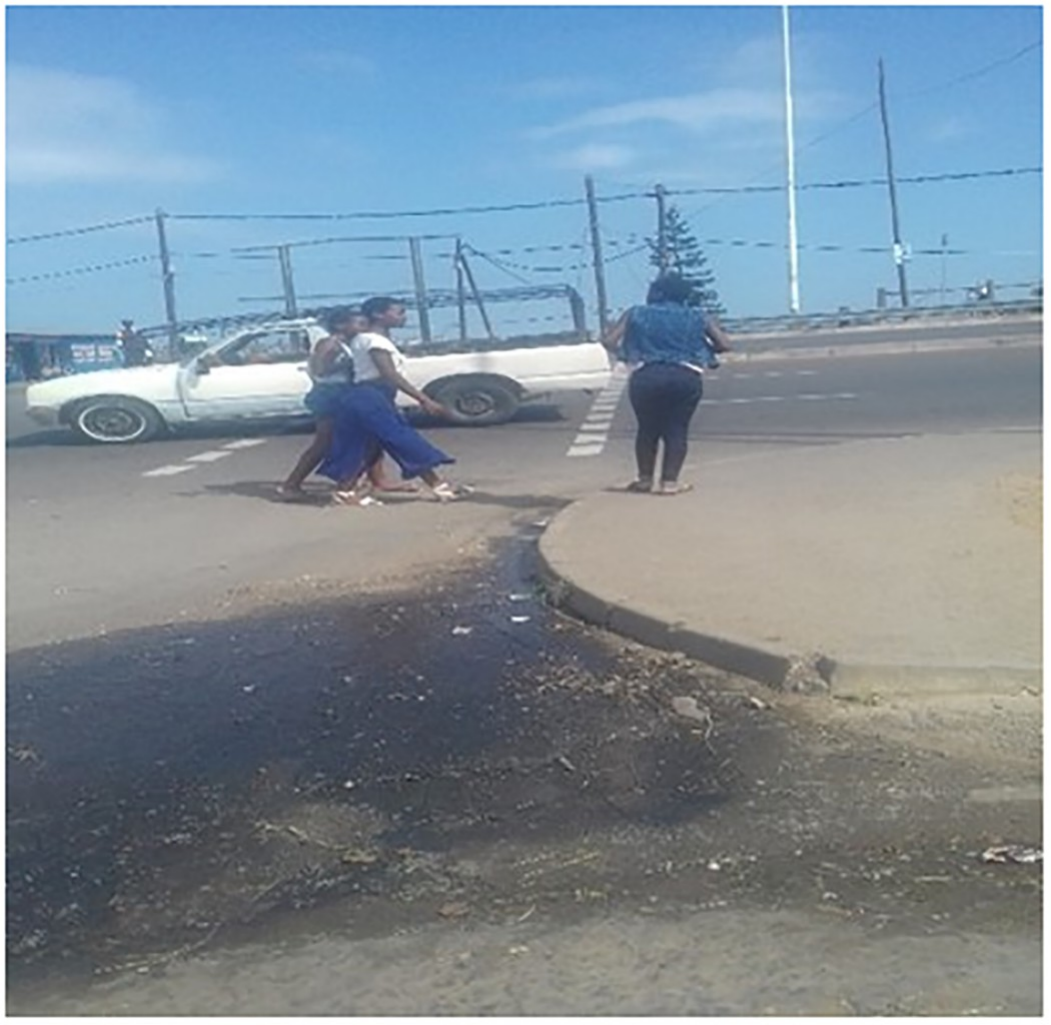
Orphaned girls standing on the street.

The second image (Fig 2) shows a group of three girls waiting on the side of the street, scouting for attention from *blessers*. In the written description of the photo, the producers suggest that “*when orphaned girls stand on the streets like this*, *they want attention and money from blessers*.”

Again, this image and description confirm an awareness of the transactional nature of relations with older ‘moneyed’ men. Girls actively seek the attention of *blessers*, suggesting a level of agency from them, as they know that older men will be attracted to them and will pursue a transactional relationship with them.

We opened this paper with Lwandle’s narrative which suggested that her sexual encounter with an older male partner was unwanted. In particular, Lwandle’s assertion that “*I end up sleeping with him even though I don’t want to”* suggests sexual manipulation, exploitation and coercion; even though she does not directly name her predicament as such. This talking in context without directly naming sexual violence is common practice in discussions about sexual exploitation and abuse in South Africa and elsewhere (see, for example, [52, 53]. For example, the often-used phrase ‘sex with a minor’ really refers to statutory rape [54]. The adolescent girls in this study also used euphemisms and code language to discuss the *blesser/blessee* dynamic. For example, none of the girls referred to their experiences as sexual violence. Instead, they suggested that *blessers* “*sexually used us*.” We discuss the key findings from our analysis of the focus group below.

#### Family neglect, survival sex and the need for material gain

Our findings affirm the notion of survival sex; a phenomenon that describes the “exchange of sex for material goods (food, shelter, sanitation, etc.) required for survival” [40]. Czechowski and colleagues argue that sexual exchange occurs under conditions of deprivation where young women exchange sex for material goods and economic security. Our participants described deprivation and neglect in their families and, in some cases, expectations from families to provide for themselves, as conditions that pushed and pulled them towards relying on older men for their basic needs and provisions. Under these circumstances, the girls own their participation in the transactional arrangements and are ready to accept blame for the relationship. The following excerpt from the focus group discussion illustrates:

*15-year-old Amanda: But we sometimes shift the blame on us. But sometimes we blame our families.*

*Researcher: Okay, what about families?*

*15-year-old Amanda: They taught us.*

*16-year-old Luthando: By allowing me to do whatever I want to do at home*

*Researcher: You do not have any rules at home?*

*16-year-old Luthando: Yes.*

*Researcher: So, in other words, you do not get everything you need at home?*

*Participants: Yes!*

*15-year-old Happy*: *The people I live with do not give me the love or things I need*. *It forced me to end up dating and sleeping with the blesser*.

The exchange above reveals how the systematic disempowerment of adolescent girls and sociocultural norms that position men as providers often compound orphaned girls’ need to engage with *blessers* for necessities; a form of survival sex. Elaborating on the reasons for entering a *blesser* relationship, 17-year-old Kim added:

*It does happen. You see, my (maternal) aunt that I live with gets my child support grant and never gives it to me. So, I never have money … now this is what happens: my aunt’s children insult me at home. If I try to reprimand them or try to reason with them, they say ‘Keep quiet because you are taking our money’ and that hurts me. Our caregivers greatly impact us in making these choices*

*(to date blessers)*.

The participants’ narratives suggest a form of financial neglect from their caregivers. For example, Kim points to her aunt’s neglect as a reason for her decision to engage sexually with an older man for money. Therefore, our analysis suggests that orphaned girls’ unmet basic needs undermine their capacity to refuse or negotiate survival sex.

#### Blessers at the nexus of chronic poverty

Our analysis suggests that girls receive messages from their families that they have a responsibility to support themselves or supplement where the family cannot provide amenities such as feminine hygiene products (sanitary pads, deodorant, and face wash). Sixteen-year-old Lethu, for instance, seems to have internalised the obligation to contribute to her basic needs, such as shelter, clothes, and food.

*Just look at us as girls. Like, I want my life to go on. I need to be doing something in life so that I do not go to bed hungry, to be able to clothe myself and have a place to sleep.*

Lethu paints a picture where she lacks a strong social safety net that might provide her everyday basic needs for food, clothes and shelter. Her state of precarity is not unique in this sense of responsibility and obligation. For example, 14-year-old Mbali cites starting her periods and needing a transactional relationship with a *blesser* to help provide sanitary products for her periods. She said “*It is also not wrong that I date a blesser because I want this thing and that thing*. *Maybe because I have started my periods (menstruation)*.” Fifteen-year-old Sthandwa cites the need for food as a reason since her caregivers only buy groceries once and expect them to last “*several days*”:

*I can say yes in my home we are poor. You can persevere. I can go for a week without eating. Not because I do not want to eat but because there is no food … My caregivers sometimes do not understand when you explain that we’ve run out of food, and they’ll just say “But I bought the grocery and I was expecting it to last for several days”. They forget that people carry lunch boxes to school. There are also other children at home, they end up eating everything and there’s nothing you can do.*

The excerpts above illustrate the intense individual-level pressures exerted on orphaned girls; pressures that create vulnerability to a sexual relationship with an older man to gain basic necessities such as food to survive. In Sthandwa’s case, a transactional relationship with a *blesser* seems the only means to obtain additional provisions in the context of inadequate groceries.

#### Choosing a *blesser*

In the excerpt below, 15-year-old Emma introduces ideas about agency, choice, intentionality, and decision-making on one hand, but also cautions and speaks of responsibility, accountability and blame on the other, on the part of orphaned girls who date *blessers*. There seems to be a complex entanglement between choice and the undesirable outcomes of blame from the negative outcomes of a *blesser* relationship for the orphaned girl. For example, Emma implores the girls not to choose someone who is attracting them with money or “*what he is going to give you*”. She warns that doing this will eventually lead to girls contracting HIV; claiming that *blessers* might rape them and leave them pregnant and abandoned.

*Can I please say this? Good people, to girls that have blessers! Choose the blessers that you will date wisely. Don’t go for someone that you see as a BEE (businessman). Go for someone that you see is not attracting you with their money, or with that mentality. No. Because some just want to experience what dating a young girl is like. Just know there is that mentality from some blessers*.

The paradox in Emma’s statement, which advises girls to refrain from falling for seemingly rich *blessers*, contrasts with the purpose of *blesser* relationships in the first place, which is that the men can provide for girls in exchange for sex. Since the purpose of these relationships is transactional, a *blesser* must necessarily be older and have money. Emma’s statement frames the girls as free, autonomous, and equal participants in relationships with *blessers* and, therefore, able to equally negotiate the terms of transacting and controlling the outcomes of such transactions. From this perspective, when this does not happen, and adverse outcomes are experienced, such as sexual assault, contracting HIV, unwanted pregnancy, and being abandoned, the girl is blamed for failing to choose their *blesser* wisely. In this regard, the following conversation elaborates:

*Tee: Do not be easily attracted to things. If you end up being with the BEE (businessman) because of what he is going to give you. And you will end up saying that he raped you. No! Choose your blesser wisely, do not go around dating everyone. You will eventually end up contracting HIV. Get pregnant and be a single mom. Who will support the child?*

Mbali: *That happens*.

Tee: *No*, *sometimes it is not the blesser’s fault*.

Sthwanda: *It is not their fault*.

Tee: *Sometimes it is our fault*.

From this conversation, the girls seem to absolve the *blessers* from blame or responsibility for their actions by framing the decision to enter transactional relationships as independent, voluntary, and free of coercion or unequal power influences; girls are spoken of as having decision-making powers regarding who to date and whether, when, and how to have sex with their *blessers*. Available scholarship contradicts these sentiments. For example, a study that explored the ways young women constructed their femininities and exercised agency in the resource-poor communities of the Eastern Cape province in South Africa [55] acknowledged that these women appear to have considerable agency at the point of choosing a sexual partner. However, once the choice was made, women’s “power was greatly circumscribed, and in many respects surrendered” (p. 8).

The girls’ narratives are notably silent on the role and power of the *blesser* and how these men may be blamed for unbecoming behaviours or unwanted outcomes of the relationship. The perspectives and silences in these discussions reveal an internalised heteropatriarchal value system among the participants in which men’s behaviours are normalised and exempted from scrutiny and in which girls and women carry the burden of blame and criticism [41].

#### Acquiescence to *blesser* expectations

These unrealistic assumptions of their power in a transactional arrangement with a *blesser* are ironically linked with the girls’ sense of obligation to unconditionally fulfil all and any sexual favours for the men. As research elsewhere has shown [23], orphaned girls express culturally informed acquiescent femininity where they surrender power to older men under the auspice of ‘choice’ to sustain their socioeconomic lives. Kim, for example, explains:

*If he continuously gives you money, you should know that nothing is for free. I can’t always give you money. Okay, maybe that can happen sometimes where maybe a person is giving money for charity or when they’re just sincerely giving money to someone. Whenever someone is expecting you will give them money, some days, I will give you money and you give me sex. That is what blessers say, ‘You spend my money and I’ll have sex with you’.*

For Kim, this is survival sex: the expectation for sex is warranted by her accepting money from a *blesser*. Within this context, sexual terms are dictated by the person with financial power. Again, we see the deprivation of basic needs on the part of orphaned girls limiting their capacity to negotiate or freely consent to survival sex.

Similarly, for 16-year-old Nati below, when a girl visits a *blesser* in his home, there should be no reason for her to refuse him sex. Also, the refusal of sex between people who are dating is precluded, indicating the belief that once a girl enters a transactional or sexual relationship, she can no longer negotiate terms of engagement within that arrangement. Nati elaborates:

*There is no need for you to not want to sleep with him if he asks you because you are like people who are dating. Because you will keep visiting him at his place and having a conversation. Because you have different needs.*

Confirming a *blesser’s* unconditional and irrefutable right to sex with the *blessee* “*no matter what*,” 15-year-old Happy notes:

*You see as girls we tend to look at things from different perspectives. Because if he were to do that to me, I would always expect that this guy who is always giving me money, one day he will want something in return that he would want me to do for him. No matter what it is I will have to try by all means to do it for him because he would be not raping or abusing me at all. We are like people who are dating.*

For Happy, once a girl accepts money from their *blesser*, they are unconditionally obligated to return sexual favours. As highlighted in much of the literature on transactional sex, this perspective further exposes girls to sexual exploitation [22, 51, 52]. Instead, participants here suggest that orphaned girls in transactional relationships must shoulder the consequences of their sexual encounters. Tee was adamant:

*Yes, good people, I say oh my lord. Let us not blame the blessers. What I am saying is that if you see you are in need do not look at the blessers as your solution. Then when you are pregnant and have contracted HIV want to blame the blesser. No, let us not do that.*

In the excerpts above, orphaned girls sit at the receiving end of heteropatriarchal power that renders them defenceless in terms of negotiating their heterosexual relationships with older men. For these participants, once one accepts money from a *blesser*, they have no right to refuse sex or negotiate the terms of engagement. By implication, they cannot (or should not) report abuse or blame the *blesser* for the negative consequences they might suffer in the relationship. This is the case even though unequal gender and social power in these relationships preclude orphaned adolescent girls from deciding whether, when, or how to have sex. For the participants, refusal or reparation is not an option, and the *blesser* remains blameless. In the participants’ understanding, the blame for transactional sex and any negative consequences that may arise rests squarely on the girl.

#### Negotiating the terms of the relationship

The obligation, referred to above, to fulfil assumed sexual obligations, regardless of whether protection is used or not and their vulnerability to sexual abuse and exploitation are obscured by the girls’ belief that entering a transactional relationship with a *blesser* strips them of all constitutionally guaranteed rights, especially the right to safe sex and to consent or refuse sex. The following exchange with 14-year-old Amanda in the focus group discussion illustrates:

Researcher: *Can you negotiate with a blesser about having sex?*

Amanda: *You can because he is not having sex with you without your consent*. *Others do not want to use a condom*, *so we might as well just stop*.

Like Amanda, Nati asserts that young girls in relationships with blessers can negotiate the terms of sexual engagement with them:

*It happens that a blesser doesn’t want to have a child with you because you are still young. When you look at it, it is very rare to find a young girl having a baby with a blesser because blessers know that their job is to sleep with you using a condom. But sometimes others use contraceptives. Others say, “They do not like to use a condom, and can’t feel anything when using a condom.” But you just explain to your blesser until he understands that you must use a condom.*

Nati and Amanda suggest that there is room to negotiate terms of engagement with a *blesser*, confirming the belief that adverse outcomes of these relationships result from girls not choosing wisely or perhaps not speaking up for themselves when there is room to do so (see also, [23, 56]. Available studies present overwhelming evidence that girls in such situations do not have such power to negotiate and are, therefore, vulnerable to sexual abuse, and sexually transmitted infections, including HIV infections and unplanned pregnancies.

Indeed, the orphaned girls’ belief in their ability to reason with a *blesser* contradicts their insistence that one cannot refuse any demands from the *blesser*. The girls’ narrations of *blesser/blesse* dynamics consistently reveal the lack of power to negotiate, a strong sense of obligations to fulfil *blesser* demands, and an inability to hold these men accountable for their actions or to allocate blame or responsibility to them (see, also, [19, 26].

## Discussion

Over two decades ago, researchers [57] used the theory of gender and power to understand and illustrate factors that create vulnerability to HIV infection among young African-American women. Our study similarly shows that orphaned adolescent girls—who live in marginalised and resource-constrained South African township contexts—share similar economic and psychosocial factors that mirror the vulnerability of their African American counterparts. In both contexts, AGYW are prone to engaging in transactional sexual relations with older men [22]. Our analysis reveals that orphaned adolescent girls report difficulties in negotiating terms of sexual relationships with their older male partners [58]. The economic and gender power compounded by age-regulated hierarchies gives men the advantage to exercise power over orphaned girls and dictate the terms of engagement [59]. Sociocultural norms in heteropatriarchal societies advantage men (and the elderly) by ascribing a higher social status to them, thus creating a climate of deference and subordination of young women in sexual relations [2, 60].

Indeed, statistics show that black South African AGYW between the ages 15–24 have the highest rate of HIV infections, experience relentless gender-based violence, and have high rates of unplanned pregnancies [2, 58], and the theory of gender and power explains how economic dependency to older male partners enforces this vulnerability. Like sex workers, young women in *blesser* relationships are in a low-control environment with limited bargaining power in terms of transactional sex [34]. The paying party holds more power and uses it to dictate terms of engagement [61].

Indeed, Connell’s theory helps us to understand the vulnerabilities of orphaned adolescent girls in *blesser* relationships. However, participants in our study discussed strategies they use to identify, assess and attract *blessers*. Perhaps, in emphasising vulnerability factors, the theory we used does not adequately help us to explain the agency and proactive approach to *blesser* relationships the girls in the study express. While girls are often framed as inarticulate and helpless victims, the participants in this study demonstrated that they could act intentionally and as active agents to initiate a transactional relationship. For example, the girls recognised that they are priced by some men for their youth, suggesting that "*some men like to sleep with young girls*" and the potential to take advantage of such attractiveness. The girls’ narratives show that even in their vulnerability they understand the relative power they have, especially in the initial stages where it may be opportune to negotiate the terms of engagement. They were acutely aware that poverty, family neglect, and unmet basic needs led them consciously into relationships where they were disadvantaged and at risk.

So, whilst the participants’ narratives confirm their vulnerability to men’s unequal gender and power, they further give us a glimpse into the less understood area of the relative agency, intentionality and proactive approach, albeit limited, that orphaned girls in resource-constrained contexts adopt to initiate or at least negotiate *blesser* relationships. The findings further give a snapshot into the potential power girls might leverage in negotiating safer sex practices such as condom use. Further research is required to expand our understanding of girls’ agency in the context of vulnerability and gender-based violence, as this holds potential for interventions that might enable them to better advocate for themselves in the context of unequal power and sexual relations in resource-limited communities such as townships.

The South African context of gender inequity deeply influences the dynamics of relationships involving adolescent girls and young women (AGYW). Due to limited access to economic resources, AGYW, especially orphans, are often driven into transactional relationships, such as those with "blessers," where older men provide material support in exchange for sex [38, 58]. This dynamic reinforces the notion of masculinity being tied to control over economic resources and perpetuates the objectification of women’s sexuality as currency [62, 63].

To challenge these entrenched social norms, it is essential to address the underlying structural factors, such as poverty and patriarchal gender norms, that sustain these inequities. Efforts should focus on empowering AGYW economically and promoting gender equality through education and awareness campaigns that redefine masculinity and femininity. Interventions that foster economic independence among women, such as income-generating programs and financial literacy initiatives, are critical in reducing AGYW’s vulnerability to exploitation [64–66].

Evidence from similar contexts suggests that programs offering comprehensive sexual education, mentorship, and community-based interventions aimed at shifting social norms around gender and power can be effective [67, 68]. These strategies can help AGYW navigate economic challenges without resorting to transactional sex while fostering healthier gender relations that are not based on economic control.

## Conclusion

This study draws attention to the transactional nature of sexual relationships between older men (*blessers*) and orphaned adolescent girls in South Africa’s resource-constraint township neighbourhoods. We have demonstrated how the structural dimensions of their lives constrained girls’ agency. The structural dimensions included their status as orphaned girls, the operating system of heteropatriarchy, age-related hierarchies, and poverty in their households and communities. Connell’s theory of gender and power helped us to understand the constraining factors that continue to shape the lives of these orphaned girls. Yet, girls also demonstrated a level of agency in negotiating the terms of their relationships with *blessers*, a phenomenon that falls outside the scope of the theory of gender and power. Overall, this paper points to *blesser* relationships as a response to chronic poverty and neglect that encompasses the lives of township orphaned adolescent girls, highlighting the need for greater support.

## Supporting information

S1 Checklist COREQ (COnsolidated criteria for REporting Qualitative research) checklist. (PDF)

S2 Checklist Human participants research checklist. (PDF)
